# Life-threatening complications of streptococcal sepsis: a PICU contemporary series

**DOI:** 10.1186/s44158-021-00028-1

**Published:** 2021-12-14

**Authors:** M. Piastra, V. Ferrari, E. Picconi, T. C. Morena, L. Pezza, G. De Rosa, M. C. Fedele, O. Genovese, R. Onesimo, A. Tempera, P. Valentini, D. Buonsenso, F. Visconti, G. Zito, C. Benassi, G. Conti

**Affiliations:** 1 Pediatric Intensive Care Unit, Emergency Department, Fond.Policlinico Gemelli IRCCS, Roma, Italy; 2 Institute of Anesthesia/Intensive Care, Catholic University Medical School “A. Gemelli” Teaching Hospital, Rome, Italy; 3 Universita Cattolica del Sacro Cuore Facolta di Medicina e Chirurgia, Rome, Italy; 4 Pediatric Cardiology, Fond.Policlinico Gemelli IRCCS, Roma, Italy; 5 Pediatric Infectious Diseases, Department of Pediatrics, Fond.Policlinico Gemelli IRCCS, Roma, Italy; 6 Neonatal ICU, S. Camillo Forlanini Hospital, Rome, Italy; 7 Intensive Care Unit, Santobono Children Hospital, Naples, Italy; 8 Intensive Care Unit, Parma Children Hospital, Parma, Italy

**Keywords:** Streptococcal sepsis complications, Intensive care, Mechanical ventilation, Septic shock, Tissue tropism phenotypes

## Abstract

**Background:**

Life-threatening streptococcal sepsis nowadays represents an uncommon event in previously healthy infants and children. Critically ill patients suffering from severe streptococcal sepsis complications may present with pre-antibiotic era clinical pictures and require a timely clinical approach to achieve restitutio ad integrum.

**Results:**

We report a series of four patient groups affected by an uncommon life-threatening streptococcal sepsis, each of them exhibiting some distinct features. *Streptococcus Agalactiae* sepsis was associated with cerebral thrombotic/ischaemic lesions, whereas severe cardiogenic shock was prominent in the *Streptococcus Viridans* group; *Streptococcus Faecalis* and *β-hemolytic group A Streptococcus* patients mostly reported lung complications.

**Conclusions:**

Previous antibiotic treatments should not delay aggressive treatment in the intensive care setting. Early diagnostic suspicion, as well as appropriate and aggressive treatment provided within an intensive care setting are crucial for the clinical outcome.

## Background

We report a series of 15 consecutive cases of life-threatening sepsis complications in previously healthy infants/children caused by different streptococcal strains: *Streptococcus Agalactiae* (4 pts); *Streptococcus Faecalis* (3 pts); *Streptococcus Viridans* (4 pts); and *Streptococcus Pyogenes* (4 pts) (Table 1).

**Table 1:** Main patient data

Case no.	Age (m)/sex	Microbiology penicillin susceptibility	PICU presentation and complications	PICU stay (days)	Prism-II	PICU management	Outcome	
							(S/D)	GOS
A1	0.6M	GBS ++	*Septic shock, meningitis, sinovenous thrombosis,*	45	35	MV(13), HS[Dop, Epi], UFH, AT	S	4
A2	1F	GBS ++	*Septic shock, meningitis, multifocal intracranial haemorrhage*	23	32	MV (HFOV), HS[Epi, Nepi, Terlipr], PC	S	3
A3	2M	GBS ++	*Status epilepticus, meningitis, ischaemic stroke*	30	18	MV (4), AT, anticonvulsants	S	4
A4	3F	GBS ++	*Septic shock , meningitis, DIC, massive brain oedema-herniation*	35	35	MV, [Dop, Epi]	D	1
B1	0.1M	S. Faecalis +	*Sepsis, ascites, pneumonia*	45	25	MV (30), HS (Nepi, Dob,Terlipr), PC	S	3
B2	0.3M	S. Faecalis +	*Sepsis, pneumonia, RL enphysema*	24	22	MV(15) HS (dopa)	S	5
B3	22F	Str. faecalis +	*Pneumonia, empyema, ARF*	7	15	NIV(6), HS[Dop]	S	5
C1	23F	Str. Viridans ++	*Septic shock, DIC, cardiogenic shock*	10	38	MV(6), HS [Dob, Enox], aPC, FFP, AT	S	5
C2	28F	Str. viridans ++	*Pulm consolidation, Pleural empyema, ARF, HUS-TTP*	18	24	CVVHDF, PE, HS [DB], MV [10], fenoldopam	S	5
C3	29M	Str. viridans +	*Acute endocarditis, respiratory failure*	6	32	NIV(2), MV (4), HS [Dobu, Dopa]	S*	4
C4	0.5F	Str. viridans +	*Sepsis-meningitis* *Acute heart failure*	6	36	MV (3), NIV (2), HS (Dobu, Milrn)	S	4
D1	36M	Str. pyogenes ++	*ARF, airway obstruction* *Pulmonary hypertension*	10	18	MV(8), NO	S	4
D2	36F	Str. Pyogenes ++	*ARDS, pneumatocele, broncho-pleural fistula*	12	25	MV(20), single lung ventilation, HS [Dop]	S	5
D3	40M	Str. pyogenes ++	*Septic shock, ARDS, renal failure*	18	37	MV, CVVHDF, HS[Epi, Nepi, Terli, LS], PC suppl, fenoldopam	S	5
D4	19M	Str. pyogenes ++	*Pneumonia - empyema*	16	18	MV (1), NIV (10)	s	5

*Prism-II* is a scale that evaluates the pediatric risk of mortality in the ICU. *GOS* (Glasgow outcome scale) evaluates the degree of recovery at 3 months of patients with brain injuries

Differently from multi-resistant Gram-negative bacteria, antibiotic susceptibility did not represent the main problem to deal with. Immunosuppressed or onco-hematological patients are increasingly admitted to pediatric ICUs, while severe sepsis/septic shock in previously healthy children is decreasing in developed countries [1]. Though rarely recorded, compared to the past, streptococci-infected immunocompetent patients may require PICU admission because of serious life-threatening conditions.

## Results

### Streptococcus Agalactiae (Table 1; A1–4)

**Fig. 1:**
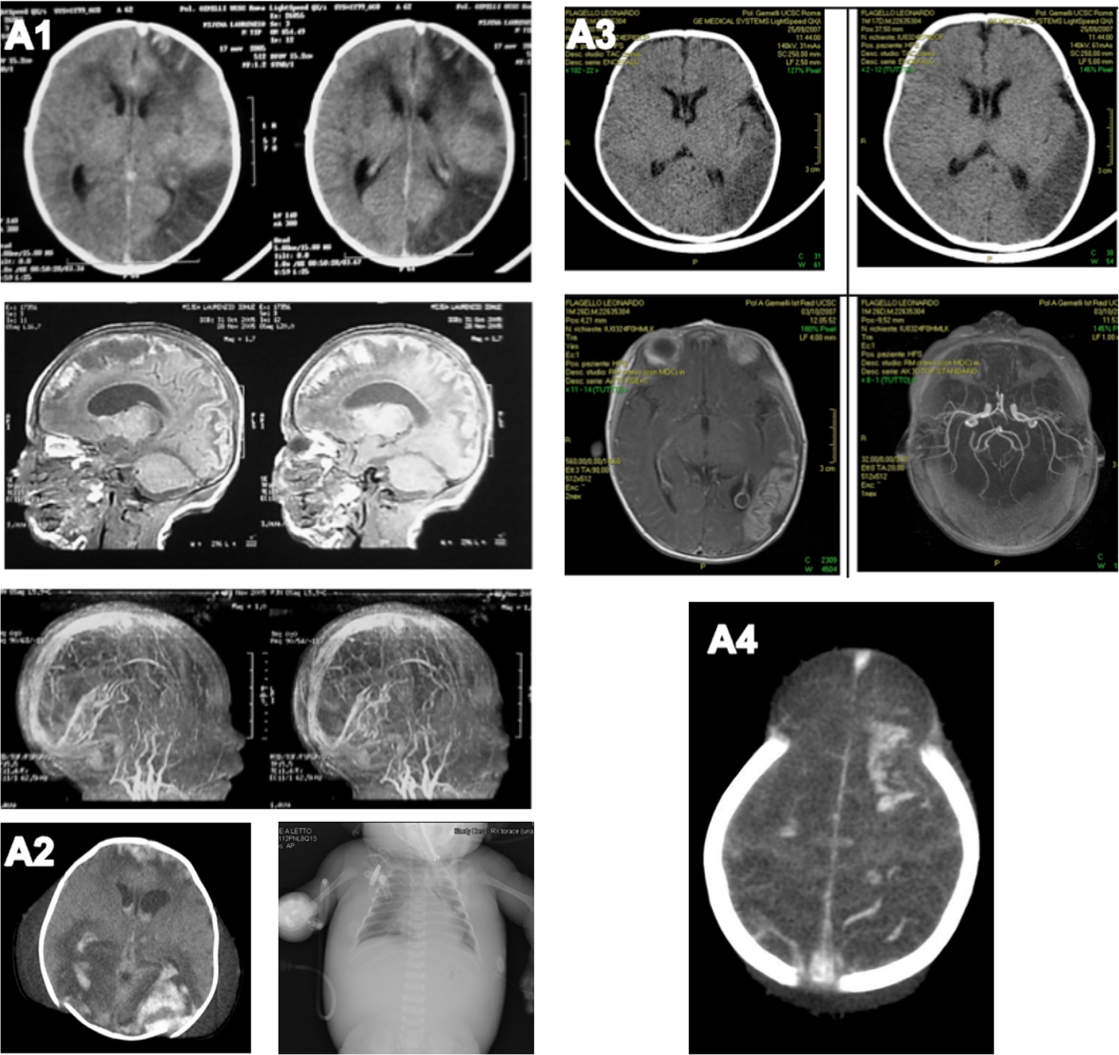
**A**–**D** Brain imaging showing different patterns of cerebrovascular damage

Patients with *S. Agalactiae* (group B Streptococcus, GBS) sepsis showed a marked cerebrovascular tropism, consistent with small vessel inflammatory involvement. Brain imaging showed different patterns of cerebrovascular damage (Fig. 1; A1–4), including severe ischaemic lesions. In case A1, a cerebral CT scan documented multiple cortical and subcortical ischaemic lesions in the left frontoparietal regions; marked brain edema was also present. A subsequent brain MRI demonstrated an almost complete thrombosis of the superior sagittal sinus with a serious compromise of the entire left cerebral hemisphere. In patient A3, a CT scan showed large ischemic cortical and subcortical lesions in the left temporal region, consequent to thrombosis of the middle cerebral artery.

None of these cases reported a positive family history for thrombophilia, and congenital hypercoagulability screening (protein C and S levels, homocysteine level, genotyping for factor V Leiden and prothrombin 20210GA mutation) resulted negative. Antiphospholipid antibodies panel was negative too, as well as the organic and aminoacidic screening.

High level respiratory and hemodynamic support was required in three patients. CSF cultures grew *S. Agalactiae*, fully susceptible to common antimicrobials. In three of the four described cases, despite multiple lesions, the clinical and neurological outcome was very favourable, whereas one infant (A4) developed marked cerebral edema and brain tissue herniation outside the cranial vault, due to which the parents asked for withdrawal of care.

### Streptococcus Faecalis (Table 1; B1–3)

Patients with *S. Faecalis* sepsis showed a marked pulmonary tropism in our experience. Three infants were consecutively admitted to our PICU, presenting with high-grade fever, tachypnea and worsening dyspnea. Standard chest X-rays and lung ultrasound showed alveolar consolidations and pleural effusions. Microbiologic analysis of samples obtained from the lower respiratory tract and blood cultures revealed *S. Faecalis* infection. Patient B3, because of documented massive pleural effusion, underwent VATS procedure and non-invasive ventilation, while younger patients underwent intubation and ventilatory support due to severe upper airway obstruction (cystic lymphangioma) (Fig. 1B). Respiratory support together with combination antibiotic therapy successfully resolved both pulmonary and systemic symptoms in all cases.

### Streptococcus Viridans (Table 1; C1–4)

Patients with *S. Viridans* sepsis showed a marked cardiovascular tropism. This patient subgroup presented with cardiovascular dysfunction requiring immediate haemodynamic support: tachycardia with severe hypotension, as well as external and internal jugular vein enlargement with increasing central venous pressure (CVP) were demonstrated on admission.

Standard chest radiographs revealed cardiac shape enlargement, and a right pleural effusion was also documented in case C1. 2D cardiac ultrasound revealed a severe global hypokinesia with a low ejection fraction (EF) despite fluid challenge and inotropic support with dobutamine. Enoximone was added to support cardiac contractility and a gradual improvement of the cardiac function was observed in the following 72 h, with increasing EF up to 50%. Cardiac enzymes (especially troponin and CK-MB) were increased, reflecting cardiac injury consistent with myocardial stunning. After blood cultures, a broad-spectrum antibiotic therapy was started including ceftriaxone and ampicillin. Purpuric lesions were noticed on the skin, mimicking a meningococcal infection (Fig. 1C). A severe coagulation impairment was also documented evolving into overt DIC together with a severe protein C deficiency. Fresh frozen plasma, antithrombin and activated protein C were given, achieving progressive improvement.

Case C3 presented a febrile illness with hypotension and heart systolic murmur. After 36 h, the cardiorespiratory status worsened: heart murmur evidence was increased, and CVP was high despite diuretic treatment. A 2D cardiac ultrasound showed hyperechogenic findings of the mitral valve. A moderate-to-severe valve insufficiency was documented and pre-existing congenital mitral valve cleft was hypothesised. Moderate pulmonary hypertension together with tricuspid regurgitation was also present. The child was referred to the heart surgery department after 6 days from PICU admission.

Case C4 presented as sudden severe heart failure, thereby revealing a true meningitis-septic shock picture: dobutamine and milrinone were given to improve myocardial contractility.

### Streptococcus Pyogenes. (Table 1; D1–4)

These patients showed both respiratory and systemic involvement: they were consecutively admitted to PICU for high-grade fever and worsening dyspnea.

Case D2 underwent tracheal intubation due to severe respiratory failure. No breath sounds were audible on the left hemithorax. A chest X-ray revealed a large pleural effusion; after drainage, respiratory conditions progressively deteriorated. The patient developed bilateral lung consolidations, anterior left pneumothorax and bronchopleural fistula, all of them refractory to different ventilatory strategies. Due to relapsing air-leaks, a single-lung ventilation mode was undertaken, achieving a progressive clinical and respiratory recovery (Fig. 1D).

In case D3, a young child presented with refractory hypoxemia and hypotension, evolving into cathecolamine-resistant septic shock. High-level vasoactive support was required, due also to marked LV function impairment, including multiple inotropic drugs. Very low PC levels were detected (8% nv) with peripheral ischemic lesions, requiring IV replacement. A 7-day course of CVVHDF was introduced; the child improved and could be discharged from PICU on day 12.

## Methods

All children admitted to PICU of a university-affiliated teaching hospital [Policlinico Universitario Agostino Gemelli] between 2000 and 2015 with laboratory-proven streptococcal sepsis/meningitis were included. Children with streptococcal isolates were identified by using the principal author’s database that registered every PICU admission. Age, length of PICU stay, PRISM-II, complications, GOS, and mortality were recorded. Ethics approval for this review was obtained from the Clinical Research Ethics Committee of our University. The bacteria were identified using conventional diagnostic methodology from specimens including blood cultures, cerebrospinal fluids, pleural aspirates, and tracheal aspirates. The diagnostic procedures were performed in the same microbiology laboratory with standardised procedures and equipment.

## Discussion

We present 15 cases of streptococcal sepsis complication treated in a PICU setting. Pneumococcal sepsis patients have not been considered in this series. The rarity of the clinical presentation warrants comments regarding both the occurrence and the intensive care approach. Epidemiological and intensive care management issues are discussed on a group-by-group basis.

Regarding *Streptococcus agalactiae* (GBS), following the CDC 2002 Guidelines recommending a universal screening for maternal rectovaginal GBS colonisation at 35–37 weeks of gestation, a clear change in the epidemiology of *S. Agalactiae* occurred, with a marked decrease in the prevalence of early onset GBS disease (2.0 per 1000 live births in 1990; 0.6 per 1000 by 2001–2002; 0.3 per 1000 in 2004) [2–4]. On the other hand, only a mild modification of the late-onset disease incidence was registered during the years 1996–2004 in the UK and, for the first time in 2003, the rate of late-onset disease overcame the one of early-onset disease [5, 6]. Cerebrovascular complications induced by *S. Agalactiae* late-onset sepsis are not well described, though an increased prevalence of meningitis is acknowledged. The occurrence of cerebral arteries thrombotic/ischaemic lesions and cerebral venous sinuses thrombosis in association with a purulent meningitis/septic shock have been described elsewhere, but they were rarely caused by neonatal *Streptococcus agalactiae* (group B streptococcus, GBS) infection, as in the present series. A rapidly progressive thrombotic occlusion of cerebral venous sinuses is associated with fatal cerebral vascular tamponade. Clinical suspicion is of paramount importance, while head CT scan may result falsely negative in up to 16% of patients [7]. The emergency intervention is aimed at maintaining venous outflow patency, despite the impending risk of intracranial bleeding. Despite the risk of further hemorrhagic complications, intravenous unfractionated heparin (UFH) anticoagulation in association with antithrombin replacement was performed because of severe compromised cerebral condition. The neonate also needed cardiorespiratory support to improve cerebral perfusion. Protein C, protein S and antithrombin deficiency are known causes of thrombosis. However, it is more likely that depressed levels of protein C, protein S and antithrombin in our patient were related to the early consumption of these factors by the acute cerebral venous infarction and/or by the septicaemic state, rather than a cause of thrombosis. No randomised controlled trials in infants and children could support such a therapeutic choice, and even in adults there is no clear evidence [8, 9]. Consequently, despite the cerebral venous bleeding, a schedule of UFH followed by a 3-month course of low-molecular-weight heparin (LMWH) was given [7, 10]. In a large multicenter Canadian study, the incidence of cerebral sinovenous thrombosis (CSVT) was 0.67/100,000 children/year, 43% of whom were under 1 month of age; CSVT may cause cerebral venous infarction and hemorrhage because of occluded venous outflow. As a whole, prothrombotic risk factors are present in 39 to 54% of cases.

Regarding *Streptococcus faecalis*, literature highlighted a rising incidence of enterococcal infections during the last years, not only among adult patients but also among hospitalised patients of neonatal/paediatric intensive care and onco-haematological units [11–14]. Although many genetically different subtypes have been identified, *E. faecalis* and *E. faecium* represent the two main species causing most human enterococcal infections. These agents represent a large group of gram-positive natural inhabitants of the human gastrointestinal tract: possible clinical presentations of enterococcal infection include intra-abdominal infections, endocarditis, primary bacteremia, urinary tract infections and, less frequently, pneumonia, meningitis and osteomyelitis (especially in immunocompromised hosts). Only a few cases of *E. faecalis* thoracic empyema have been reported in adults. The occurrence of enterococcal sepsis followed by a pleural empyema in a previously healthy child is almost exceptional. Although enterococcal empyema is rare, its occurrence has been previously linked to intra-abdominal infections, and it is associated with a high mortality (41%) [15]. Numerous evidence shows that the majority of enterococcal-associated infections of the lower respiratory tract have a complicated course, including the development of lung abscess, pleural empyema, respiratory failure and cardiovascular instability. Therefore, *Enterococci* should be considered as potential cause of severe, unresolved pneumonia. It appears of importance to avoid a prolonged course of invasive mechanical ventilation following a high-intensity antibiotic therapy. For this reason, in the case reported above, following the VATS procedure, we decided to introduce the NIV technique early, to minimise the risk of pulmonary-systemic superinfections.

*Viridans streptococci* (VS) represent a heterogeneous group of streptococcal species, which are part of the normal flora of human oral cavity, gastrointestinal tract and female genital tract. Infections usually result from spread of the organisms outside their normal habitat. Strains of *Viridans streptococci* are known to be the most common etiologic agents in bacterial endocarditis, mainly in patients with abnormal cardiac structure [16, 17]. Immunocompromised patients with VS bacteremia may develop shock, rash and respiratory distress similar to ARDS. VS may account for up to 25–30% of bacteremic episodes in patients with malignancies; VS bacteremia should be suspected in neutropenic children, especially in the presence of mucositis [18, 19]. Viridans streptococci shock syndrome (VSSS) has been defined as hypotension requiring inotropic support [20]. VS have been frequently described as a cause of bacteremia, and VSSS develops in up to 18% of VS bacteremia in children with cancer or SCT recipients. Infection of immunocompetent individuals is generally rare [21]. Although primary pneumonia due to *Viridans streptococci* has rarely been described, published reports documented recovery of these organisms in specimens of empyema and lung abscess, whereas VS septicaemia appears exceptional [22–24]. We observed a severe cardiac involvement with septic shock and consumption coagulopathy due to VS infection. Cardiac function impairment resembling a “cardiac stunning” picture is rarely present at onset of septic shock. In the two patients reported, after failed initial preload optimisation and dobutamine titration, a inotropic rescue with enoximone/milrninone was performed. These agents selectively inhibit phosphodiesterase III isoenzyme in both cardiac and vascular muscle, which is necessary for the breakdown of 3′5′-cyclic adenosine monophosphate (cAMP) [25]. Due to the severity of clinical presentation, recombinant APC infusion was started, achieving coagulative status control [26]. Though *S. Viridans* represents a known agent of infective endocarditis, a severe sepsis complicating pneumonia with cardiac valve involvement is unusual. All patients were empirically treated with third generation cephalosporins and amikacin before susceptibility testing was available, and recovered without complications.

Concerning *Streptcoccus Pyogenes* invasive disease, an increased incidence has been reported during the last years [27]. The incidence of invasive *S. Pyogenes* disease in Italy is 0.38/100,000 (3/100,000 in Europe), with most cases among older patients (only 24% of cases in the 0–17 years old group) [28]. Overall, 8% of patients with *Streptococcus Pyogenes* infection develop necrotizing fasciitis and 13% evolve to streptococcal toxic shock syndrome (STSS), with an incidence rising to 50% among cases of necrotizing fasciitis. The only clinical manifestation showing a higher prevalence in children < 10 years old is meningitis (2% of overall cases). Invasive group A Streptococcus (GAS) infections have been reported in all age groups, including sepsis, bacteremic pneumonia, puerperal sepsis, septic scarlet fever, scarlatina maligna, erysipelas, necrotizing fasciitis, gangrene, myositis and streptococcal toxic shock syndrome [29–31]. A rapid and fatal course has been described even in the immunocompetent host, with a mortality rate of 7-58% [32]. Several risk factors have been associated with invasive GAS infection, in particular VZV co-infection and the use of nonsteroidal anti-inflammatory drugs [33]. Though invasive GAS infections are alarmingly increasing worldwide, an acute respiratory distress syndrome caused by GAS has been rarely described in pediatrics. Despite non-staphylococcal pneumatoceles being described in childhood, reports of GAS pneumatoceles are extremely rare. In our case, a GAS pneumonia and pleural empyema rapidly progressed to ARDS. Pneumothorax and a broncho-pleural fistula complicated the ventilatory management. Failing of conventional therapies (gentle conventional ventilation, surfactant instillation) led to the adoption of a single lung ventilation. Apart from surgical indications, lung isolation may be appropriate even in unilateral lung disease (e.g., lung abscess, bronchopleural fistula, severe one-sided bullous disease, pulmonary hemorrhage) [34–36]. In children, due to the small tracheal dimensions, balloon-tipped bronchial blockers, wire-guided endobronchial blocker, UniventTM tubes, double-lumen tubes and selective mainstem bronchial intubations with a conventional endotracheal tube may be chosen [37–39]. Single lung ventilation is often complicated by the persistent collapse of the unventilated lung and by the obstruction of the right upper lobe in case of right-sided intubation. For these reasons, only time-limited applications can be recommended. In our case, a 36-h single lung ventilation resulted a safe procedure and allowed bilateral lung ventilation restoration. Unilateral lung disease represents a challenge in ventilatory management because of the asymmetry in lung mechanics. Conventional support may fail to produce adequate gas exchange and may cause further deterioration. In those circumstances, independent lung ventilation allowing different mean pressures in each lung may represent a valid strategy. Alternatively, the most compromised lung may be treated with HFOV, while the contralateral is conventionally supported. In children, due to the small tracheal dimensions, unilateral lung ventilation may represent a valid alternative.

## Conclusion

Streptococcal sepsis remains diffusely present among the pediatric population, while severe and life-threatening cases have become consistently rarer in developed countries. Even in previously healthy infants/children, in the absence of antibiotic-resistant streptococcal species, a rapidly evolving picture of severe sepsis and septic shock may occur. Emergency physicians and intensivists should be aware of these uncommon complications, which require a timely cardiorespiratory support as well as high-dose intravenous antimicrobials. As in the presented experience, only a highly individualised approach could achieve a significant improvement of the patient’s clinical status and thereby result in a favourable outcome.

## Data Availability

The datasets used and/or analysed during the current study are available from the corresponding author on reasonable request.

## Abbreviations

ARDS: Acute respiratory distress syndrome
cAMP: Cyclic adenosine monophosphate
CK-MB: Creatine kinase-MB
CSF: Cerebrospinal fluid
CSVT: Cerebral venous sinus thrombosis
CVP: Central venous pressure
CVVHDF: Continuous veno-venous hemodiafiltration
CT: Computed tomography
DIC: Disseminated intravascular coagulation
EF: Ejection fraction
GAS: Group A streptococcus
GBS: Group B streptococcus
GOS: Glasgow outcome scale
HFOV: High-frequency oscillatory ventilation
ICU: Intensive care unit
LMWH: Low molecular weight heparin
LV: Left ventricle
MRI: Magnetic resonance imaging
NIV: Non-invasive ventilation
PC: Protein C
PICU: Pediatric intensive care unit
PRISM-II: Project Resources Information System for Management II
STSS: Streptococcal toxic shock syndrome
UFH: Unfractionated heparin
VATS: Video-assisted thoracoscopy
VS: Viridans streptococci
VSSS: Viridans streptococcal shock syndrome
VZV: Varicella zoster virus
